# Supplementary material for: Fast and global detection of periodic sequence repeats in large genomic resources
Source: Nucleic Acids Res. 2018 Oct 10;47(2):e8. doi: 10.1093/nar/gky890 (PMC6344855; doi:10.1093/nar/gky890)
Supplement: Supplementary Data [file gky890_supplemental_files.zip › SPADE_NAR_SupplementaryFigures_rev1_09182018_NY1.pdf]

Supplementary Figures for

Fast and global detection of periodic sequence repeats in large genomic resources

Hideto Mori,[1–3] Daniel Evans-Yamamoto,[1–3] Soh Ishiguro,[1–3] Masaru Tomita,[2–4] and Nozomu Yachie[1–3,5,6,*]

[1] Synthetic Biology Division, Research Center for Advanced Science and Technology, The University of Tokyo, Tokyo 153-8904, Japan.
[2] Institute for Advanced Biosciences, Keio University, Tsuruoka 997-0035, Japan.
[3] Systems Biology Program, Graduate School of Media and Governance, Keio University, Fujisawa 252-0882, Japan.
[4] Department of Environment and Information Studies, Keio University, Fujisawa 252-0882, Japan.
[5] Department of Biological Sciences, School of Science, The University of Tokyo, Tokyo 113-0033, Japan.
[6] PRESTO, Japan Science and Technology Agency (JST), Tokyo 153-8904, Japan.

* To whom correspondence should be addressed. Tel: +81-3-5452-5242; Fax: +81-3-5452-5241; Email: yachie@synbiol.rcast.u-tokyo.ac.jp

LIST OF CONTENTS

Figure S1. Conceptual diagram of the SPADE workflow.
Figure S2. Large DNA repeats found in the *Xoo* PXO83 genome with a repeat unit size of 787 that overlaps with three hypothetical genes.
Figure S3. Type III secretion system effector protein with periodic repeats.
Figure S4. Comparison of the performance of SPADE, XSTREAM, and T-REKS with various PRSs.
Figure S5. PPR-like proteins captured by SPADE.
Figure S6. Periodic tRNA operons captured by SPADE.
Figure S7. Analysis of simulated degenerate repeats using SPADE.

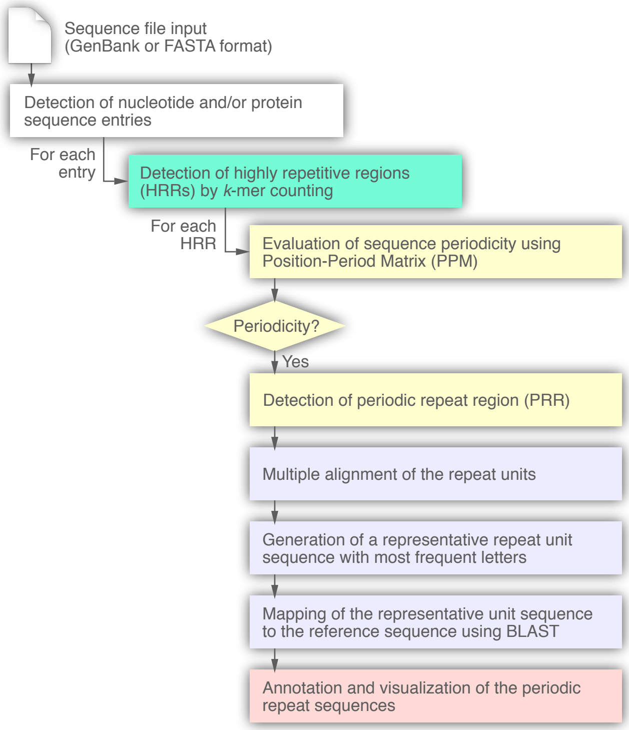

Supplementary Figure S1. Conceptual diagram of the SPADE workflow

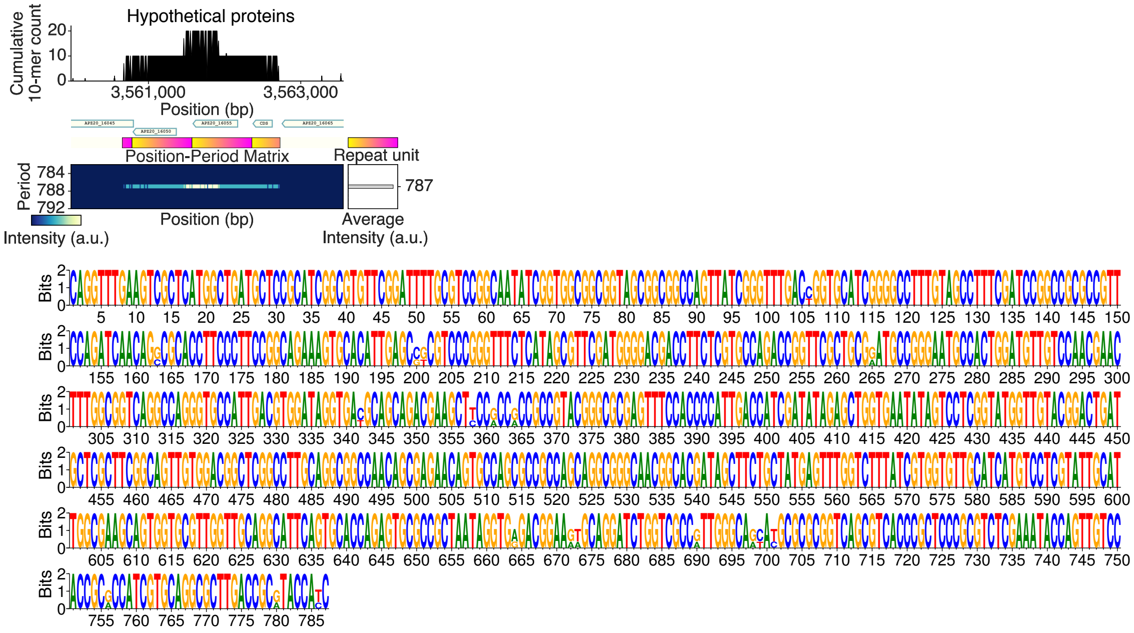

Supplementary Figure S2. Large DNA repeats found in the *Xoo* PXO83 genome with a repeat unit size of 787 that overlaps with three hypothetical genes. For details, see Figure 1B.

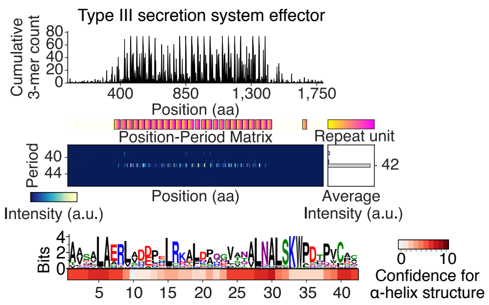

Supplementary Figure S3. Type III secretion system effector protein with periodic repeats. For details, see Figure 1B.

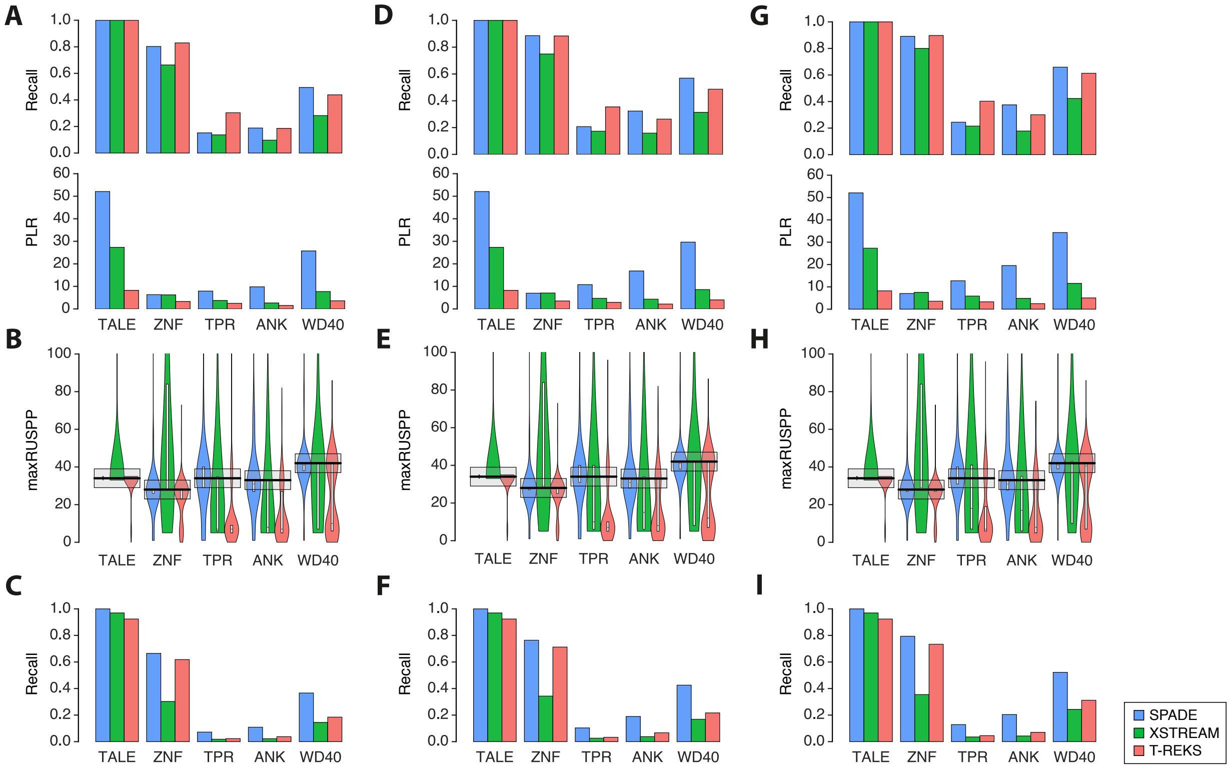

Supplementary Figure S4. Comparison of the performance of SPADE, XSTREAM, and T-REKS with various PRSs. For each of the TALE, ZNF, TPR, ANK repeat, and WD40 repeat families, different PRSs were prepared so each PRS protein involved ≥5 (**A-C**), ≥7 (**D-F**), or ≥9 (**G-I**) corresponding PRS domains. For details, see Fig. 5.

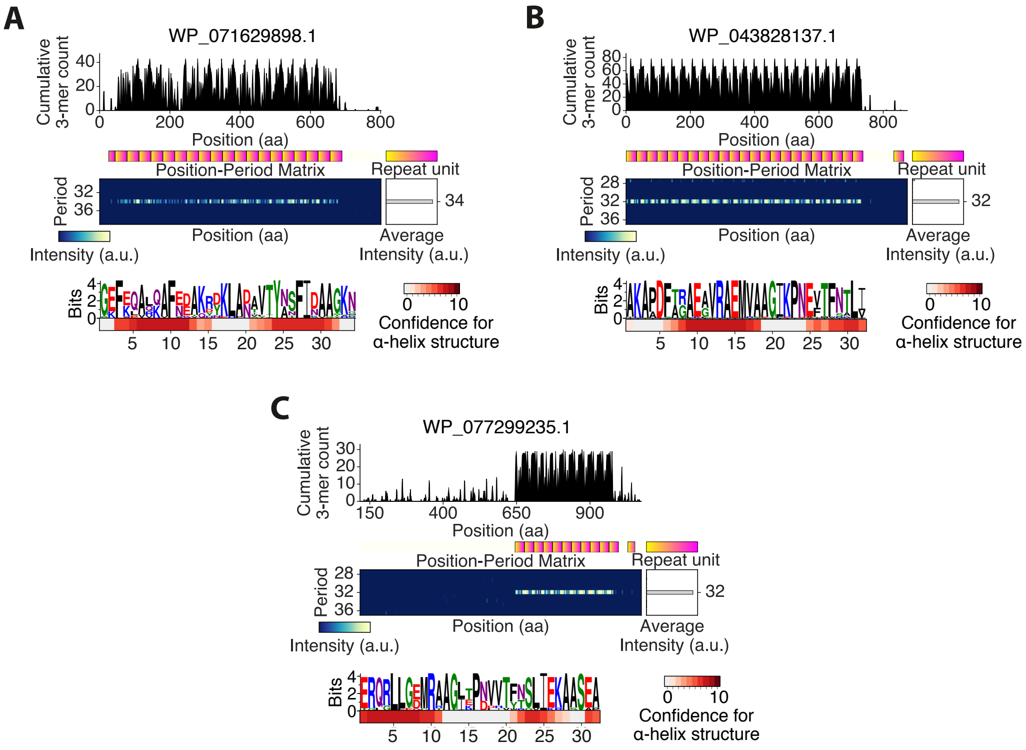

Supplementary Figure S5. PPR-like proteins captured by SPADE. From the 7,006 prokaryotic genomes, three PPR-like proteins were found to have 10 or more periodic repeats. (**A**) WP_071629898.1 from *Francisella sp*. MA067296. (**B**) WP_043828137.1 from *Aquaspirillum sp.* LM1. (**C**) WP_077299235.1 from *Rhodobacter sphaeroides* ATCC 17029. For the details of the figure representation, see Fig. 1B.

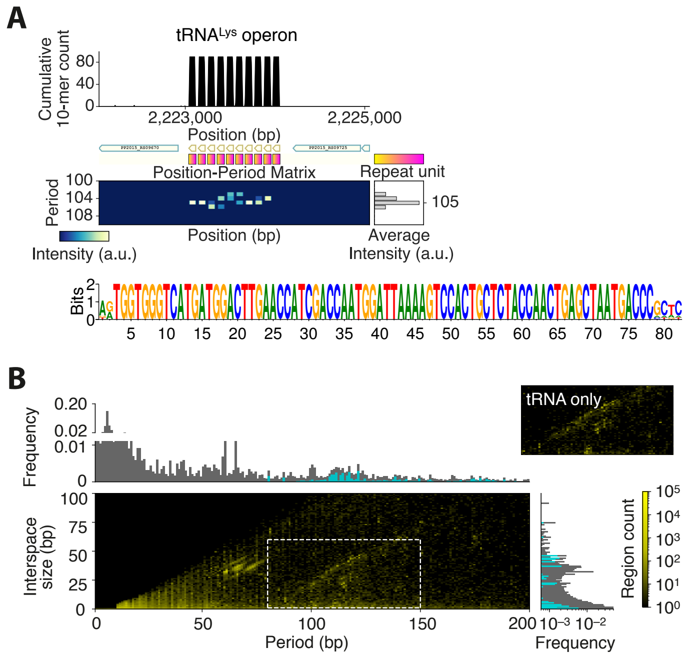

Supplementary Figure S6. Periodic tRNA operons captured by SPADE. (**A**) Example of tRNA^{Lys} operon captured in the *Pseudoalteromonas phenolica* KCTC 12086 genome. (**B**) Period-interspace size distribution of the entire periodic DNA repeats captured in the 7,006 RefSeq prokaryotic genomes with tRNA operons highlighted by a dashed white box and light blue bars in the frequency distribution diagrams.

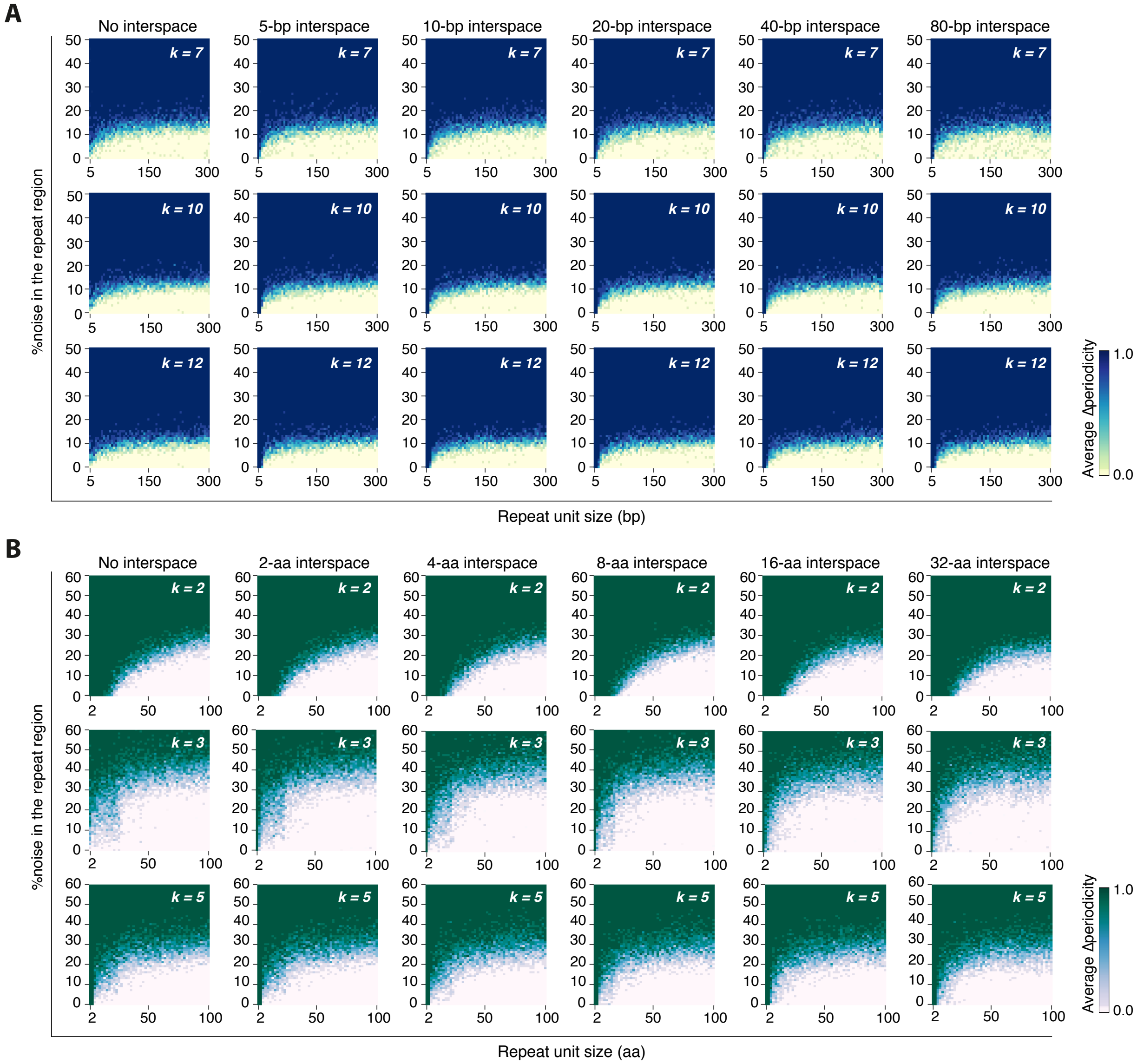

Supplementary Figure S7. Analysis of simulated degenerate repeats using SPADE. For each parameter combination of repeat unit size, interspace size, and percent noise introduced in the repeat sequence region, 10 random sequences of five repeat units with flanking random sequences (5,000 bp for DNA and 500 aa for protein) were generated and analyzed by SPADE with different *k*-mer parameters. For each simulated sequence, the difference between captured and expected repeat periodicities (Δperiodicity) was calculated as $| P_e – P_d | / P_e$, where P_e and P_d are expected and detected periodicities. (**A**) Simulated DNA repeats. (**B**) Simulated protein repeats.
